# Drug Delivery of Gelatin Nanoparticles as a Biodegradable Polymer for the Treatment of Infectious Diseases: Perspectives and Challenges

**DOI:** 10.3390/polym15214327

**Published:** 2023-11-05

**Authors:** Osama A. Madkhali

**Affiliations:** Department of Pharmaceutics, College of Pharmacy, Jazan University, Jazan 45124, Saudi Arabia; omadkhali@jazanu.edu.sa

**Keywords:** drug delivery, gelatin nanoparticles, infectious diseases, biodegradable polymer

## Abstract

In recent years, there has been a growing interest in the use of gelatin nanoparticles (GNPs) for the treatment of infectious diseases. The inherent properties of these nanoparticles make them attractive options for drug delivery. Their biocompatibility ensures that they can interact with biological systems without causing adverse reactions, while their biodegradability ensures that they can break down harmlessly in the body once their function is performed. Furthermore, their capacity for controlled drug release ensures that therapeutic agents can be delivered over a sustained period, thereby enhancing treatment efficacy. This review examines the current landscape of GNP-based drug delivery, with a specific focus on its potential applications and challenges in the context of infectious diseases. Key challenges include controlling drug release rates, ensuring nanoparticle stability under physiological conditions, scaling up production while maintaining quality, mitigating potential immunogenic reactions, optimizing drug loading efficiency, and tracking the biodistribution and clearance of GNPs in the body. Despite these hurdles, GNPs hold promising potential in the realm of infectious disease treatment. Ongoing research and innovation are essential to overcome these obstacles and completely harness the potential of GNPs in clinical applications.

## 1. Introduction

A key global health challenge today is the escalating problem of drug resistance in the treatment of infectious diseases, leading to a substantial increase in morbidity and mortality rates worldwide. According to the World Health Organization, infectious diseases such as lower respiratory infections, HIV/AIDS, diarrheal diseases, and tuberculosis are among the leading causes of global mortality, demonstrating their widespread prevalence [1]. These diseases can be caused by different types of pathogens, including bacteria, viruses, fungi, and parasites. Bacterial infections, for instance, are caused by single-celled organisms that can exist independently or parasitically, causing harm to their host [2]. Streptococcal infections, caused by a group of bacteria known as Streptococcus, can result in a range of conditions, from mild ones such as strep throat to more severe ones such as necrotizing fasciitis or rheumatic fever [3]. Urinary tract infections (UTIs), primarily caused by *Escherichia coli* and *Staphylococcus saprophyticus* bacteria, typically affect the urinary system, including the kidneys, bladder, and urethra [4]. Notably, tuberculosis, a severe respiratory disease caused by *Mycobacterium tuberculosis*, remains a significant global health problem. It often affects the lungs and can potentially impact other parts of the body [5]. Additionally, *Staphylococcus aureus*, a bacterium typically present in the upper respiratory tract, can cause infections of varying severity. These can range from relatively minor skin conditions to critical illnesses such as pneumonia, meningitis, and sepsis [6]. Similarly, *Pseudomonas aeruginosa*, an opportunistic pathogen, can cause serious infections, particularly in individuals with compromised immune systems, leading to conditions such as pneumonia, UTIs, and systemic infections [7].

In contrast, viral infections are caused by viruses, which are much smaller than cells and require a host organism for replication. HIV/AIDS, caused by the human immunodeficiency virus, debilitates the immune system by destroying disease-fighting cells, leading to the final stage known as acquired immune deficiency syndrome [8]. COVID-19, a respiratory illness caused by the SARS-CoV-2 virus, became a global pandemic in 2020, further highlighting the threat of viral diseases [9]. Another significant viral infection is hepatitis C, an infectious disease that primarily affects the liver and is caused by the hepatitis C virus. This disease can range in severity from a mild illness lasting a few weeks to a serious, lifelong illness leading to cirrhosis or liver cancer [10].

Moreover, fungal and parasitic infections represent other types of infectious diseases. Fungal infections, caused by fungi, can affect the skin or mucous membranes and, in certain cases, invade the bloodstream or internal organs. Athlete’s Foot, for example, is a skin infection on the feet caused by the fungus Tinea [11]. In addition, keratitis, which is an inflammation of the cornea, can be caused by several types of fungi, leading to vision-threatening complications if not treated promptly [12]. Candidiasis, an infection caused by more than twenty types of Candida species, can lead to oral thrush or invasive candidiasis [13,14]. Among these species, *Candida glabrata* can also cause infections, often in immunocompromised individuals, and is known for its resistance to antifungal treatment [15]. Parasitic infections, on the other hand, are caused by various parasites. Malaria, a severe disease caused by Plasmodium parasites, is transmitted by the bite of an infected Anopheles mosquito [16]. Similarly, giardiasis, an infection in the small intestine, is caused by a microscopic parasite called *Giardia lamblia* [17].

These diseases impose a substantial burden on healthcare systems and economies, particularly in low- and middle-income countries. The ongoing COVID-19 pandemic further underscores the devastating impact of infectious diseases and emphasizes the importance of robust strategies for their prevention and treatment [18].

Microorganisms, such as bacteria, viruses, and fungi, can evolve over time, developing resistance to the drugs used to treat them. This process is accelerated by factors such as the misuse and overuse of antimicrobials, poor infection prevention and control practices, and inadequate surveillance [19]. The emergence of drug-resistant strains of diseases such as tuberculosis, malaria, and gonorrhea has made treatment more difficult and increased the risk of disease transmission. Therefore, there is a pressing requirement for novel treatment approaches that can efficiently combat drug-resistant infections.

One promising approach to addressing this challenge is the use of nanotechnology in drug delivery. Nanotechnology involves the manipulation of matter at the nanometer scale to create new materials and devices with enhanced properties [20]. In the context of drug delivery, nanoparticles can be designed to deliver drugs directly to the site of infection, thereby enhancing drug efficacy and reducing side effects [21]. Moreover, nanoparticle-based drug delivery systems have the potential to overcome drug resistance by bypassing the mechanisms that microorganisms employ to resist drugs [22]. For instance, nanoparticles can be engineered to penetrate biofilms, which are protective layers formed by bacteria that can confer drug resistance [23]. Thus, nanotechnology offers new and exciting opportunities for the development of more effective treatments for infectious diseases. This review paper centers around the benefits and hurdles of using gelatin nanoparticles (GNPs) as drug delivery mechanisms for treating infectious diseases.

## 2. Gelatin and Gelatin Nanoparticles

Gelatin, a biodegradable polymer, has been gaining more interest in recent years due to its wide range of uses and eco-friendly characteristics [24]. Gelatin is also a natural and renewable substance that can be utilized in various fields, including bioengineering, pharmaceuticals, and the food industry [25,26]. Gelatin is derived from the partial hydrolysis of collagen, which is found in animal tissues such as bones, hide, and pigskin [27]. Porcine skin accounts for the majority of gelatin production at 46%, while bovine hide and bones contribute 29.4% and 23.1%, respectively [28]. A small percentage, approximately 1.5%, comes from fish [29]. There are two main types of gelatin: Type A, which is cationic and has an isoelectric point (IEP) ranging from 7 to 9, and Type B, which is anionic with an IEP of 4.8 to 5 (Table 1). The U.S. Food and Drug Administration (FDA) also considers gelatin a safe polymer [30].

**Figure 1:**
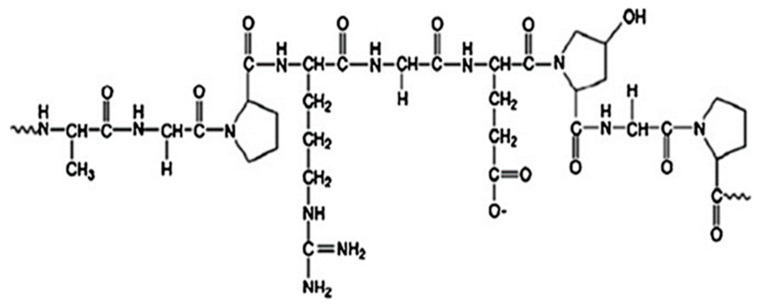
General structure of gelatin (reproduced with permission from [36]).

Comprising a mixture of peptides, proteins, and amino acids (Figure 1), gelatin possesses unique properties that make it an attractive material for various applications. It exhibits excellent biocompatibility, meaning it can interact with living tissues without causing any adverse reactions [34]. Moreover, its biodegradable characteristic allows it to naturally break down in the environment, reducing the potential for pollution and waste accumulation [35].

**Figure 2:**
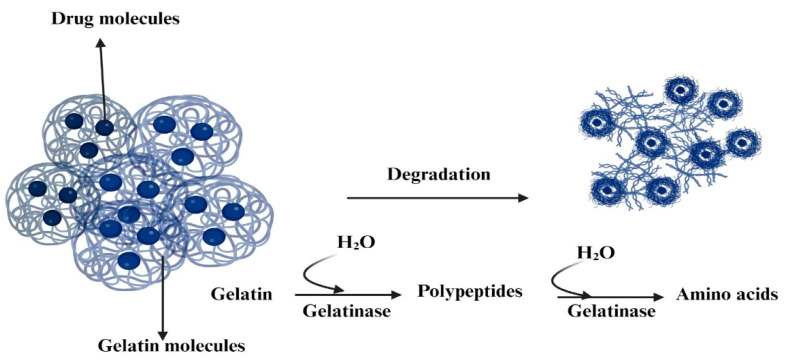
Degradation process of gelatin through enzymatic hydrolysis.

In the human body, gelatin is degraded through enzymatic hydrolysis. Multiple enzymes play a role in breaking down proteins. Protease enzymes, for instance, are capable of cleaving the peptide bonds that connect the amino acids in the gelatin molecule [37]. As a result of this process, gelatin is effectively disassembled into its individual amino acid components (Figure 2). Its biocompatibility and biodegradability make it well-tolerated by the human body, and its versatility allows for use in a range of applications, from tablet binders to coating agents [38]. Gelatin also possesses thermo-reversible gelation properties, which allow it to transition between a fluid and a gel state at different temperatures, enhancing its versatility.

One key advantage of gelatin is its ability to form hydrogels, which are networks of polymer chains capable of retaining a large amount of water [39]. The capability stems from the unique structural characteristics of gelatin, such as swelling and solubility [40]. It is composed of a sequence of amino acids that form long chains. These chains have a high proportion of hydrophilic (water-attracting) amino acids, such as glycine, proline, and hydroxyproline [41]. When gelatin is dissolved in water and then cooled, the polymer chains reorganize and form a three-dimensional network that is linked via hydrogen bonds [39,42], trapping water molecules within the structure. This process is known as gelation, and the resulting structure is called a hydrogel. Gelatin-based hydrogels are not only biodegradable and biocompatible, but they also exhibit mechanical properties similar to those of natural tissues. This makes them ideal for a wide range of applications in the environmental, medical, and pharmaceutical fields [39].

Gelatin, in comparison to other naturally derived polymers such as starch, cellulose, and chitosan, demonstrates better flexibility and mechanical strength, especially when used with cross-linking agents [43]. This makes it adaptable to a wider range of applications, including food, pharmaceuticals, biotechnology, and cosmetics. Starch and cellulose, despite being abundant and economically viable, often lack the mechanical resilience and flexibility that gelatin provides. As a result, their utilization is limited to certain applications where these properties are critical [44]. Furthermore, a distinguishing characteristic of gelatin, as opposed to cellulose, is its solubility in both aqueous and common organic solvents [40]. This feature broadens its utility, as the solubility of a polymer often predicates its adaptability in various environments and applications. Cellulose, due to its insolubility in many common solvents [45], has a more limited scope of applications. Chitosan provides excellent biocompatibility and antimicrobial properties, making it a popular choice in wound dressings and antimicrobial coatings [46,47]. However, it is less flexible and more challenging to process than gelatin, which can limit its suitability in some applications where flexibility and ease of processing are necessary [48].

Turning to synthetic biodegradable polymers, such as polylactic acid (PLA), polyglycolic acid (PGA), and polycaprolactone (PCL), each polymer exhibits a unique set of strengths and limitations. These synthetic polymers often exhibit excellent mechanical properties and durability, making them suitable for applications that require long-term stability or load-bearing capacities. However, their biocompatibility often falls short when compared to gelatin, which can limit their usage in applications involving direct contact with biological systems, such as drug delivery or tissue engineering. For instance, PCL, despite exhibiting notable flexibility and durability, is hydrophobic, making it difficult to interact with cells [49]. Furthermore, while PGA is recognized for its high mechanical strength [50], it has certain limitations that restrict its functionality in specific applications. It is insoluble in many common solvents and is characterized by a rapid degradation rate [51]. These characteristics contribute to a more limited scope in research applications, particularly in the development of PGA-based drug delivery systems [50].

The use of GNPs as drug delivery systems has exhibited multiple advantages. First, GNPs can be used to encapsulate a variety of drugs, including both hydrophilic and hydrophobic compounds. This makes them suitable for delivering a wide range of therapeutic agents. Moreover, their properties, such as size, surface charge, and drug release profile, can be easily tailored to suit specific applications by adjusting the preparation process [52]. Second, GNPs can enhance the stability of encapsulated drugs, protecting them from degradation in the physiological environment. This is particularly beneficial for sensitive drugs that are susceptible to degradation in the stomach or bloodstream, as it allows them to reach the target site in an active form [53]. Third, GNPs can be designed to target specific locations within the body for drug delivery. This can be achieved by modifying their surface with targeting ligands that bind to specific receptors on the target cells. This targeted delivery can increase the drug concentration at the target site, thereby enhancing therapeutic efficacy and minimizing side effects [52]. Fourth, GNPs can provide controlled and sustained release of the encapsulated drug. This allows for maintaining the drug concentration within the therapeutic window for an extended period, thereby improving treatment outcomes and patient compliance.

The advantages of GNPs, coupled with their ease of preparation and customizable properties, position them as a promising foundation for creating efficient and safe drug delivery systems. Current research in this area remains focused on discovering novel methods to utilize and augment these benefits, laying the groundwork for inventive treatment options [54]. For instance, in cancer therapy, GNPs can be used to deliver chemotherapeutic drugs directly to tumor cells. This approach enhances the drug’s efficacy while minimizing toxic side effects on healthy cells. Their surface can be modified with antibodies or other targeting ligands that specifically bind to receptors that are overexpressed on cancer cells, thereby improving the selectivity and effectiveness of the treatment [36,55].

GNPs have also been investigated for oral drug delivery. They can protect the encapsulated drug from degradation in the harsh conditions of the gastrointestinal tract and enhance drug absorption by increasing the residence time in the intestines. Some studies have also suggested that GNPs can enhance the oral bioavailability of poorly soluble drugs [56,57]. Furthermore, GNPs have shown promise as non-viral vectors for gene delivery. They can encapsulate multiple plasmids, and the effectiveness of the enclosed DNA can be enhanced by preventing degradation caused by nucleases. GNPs can be linked to substances that encourage receptor-mediated endocytosis [58]. Additionally, the use of long-circulating PEGylated nanoparticles can improve their bioactivity [59,60].

## 3. Preparation of Gelatin Nanoparticles

The preparation of GNPs involves several techniques, each with its own unique advantages and limitations. The four most common methods include desolvation, coacervation, emulsion, and nanoprecipitation.

### 3.1. Desolvation

**Figure 3:**
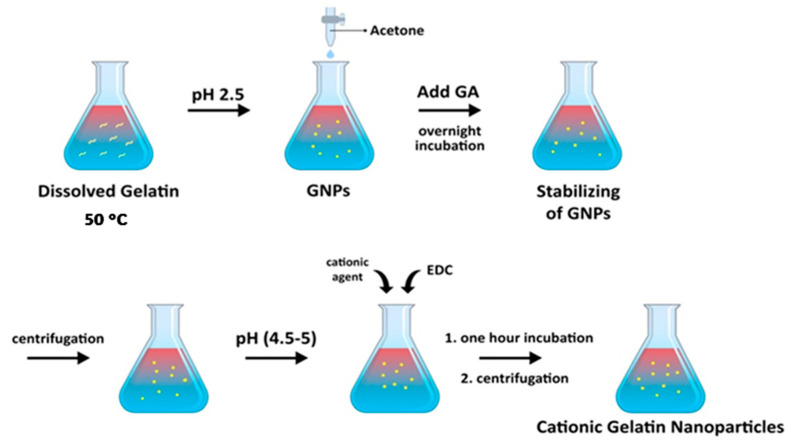
Schematic illustration of the preparation of cationic gelatin nanoparticles using the one-step desolvation method [59].

The desolvation technique is a frequently used method for producing GNPs. This method involves gradually adding a water-compatible organic solvent, such as ethanol or acetone, into a gelatin solution while continuously stirring [61]. This results in the precipitation of gelatin as nanoparticles due to the desolvation effect. The desolvation effect occurs when the organic solvent is rapidly removed from the polymer solution upon contact with the non-solvent. The drug can be added either before or during the desolvation step in order to encapsulate it within the nanoparticles. The resulting nanoparticles can be further stabilized using cross-linking agents, such as glutaraldehyde (GA) [62,63]. The resulting drug-loaded nanoparticles can provide a controlled release of the drug. The desolvation method can be conducted in one or two steps. The one-step desolvation method involves directly adding the organic solvent solution to the non-solvent aqueous solution (Figure 3) [64]. On the other hand, the two-step desolvation method involves adding a non-solvent to the organic solvent solution, followed by adding the resulting mixture to the non-solvent aqueous solution [65]. The two-step method is generally preferred because it results in the formation of smaller and more uniform nanoparticles. This is attributed to the slower and more controlled precipitation of the gelatin [59].

### 3.2. Coacervation

Coacervation, also known as phase separation, is a prevalent method for creating GNPs, which can be utilized for controlled drug delivery [66]. This technique involves two main stages: the formation of coacervates (tiny droplets) through the destabilization of a gelatin solution and the hardening of these droplets to form nanoparticles [67]. In the first stage, the gelatin solution is destabilized through changes in conditions such as temperature, pH, or adding salt or alcohol. This leads to the separation of gelatin molecules from the solution and their aggregation into coacervates. This process is influenced by several factors, including the concentration of gelatin, the rate of cooling, and the pH of the solution [31]. In the second stage, the coacervates are precipitated by adding coacervating agents such as ethanol or propanol [68] to form nanoparticles. This is typically achieved by cross-linking the gelatin molecules using a cross-linking agent such as GA [62,63] or genipin [69]. The drug can be mixed with the gelatin solution before the coacervation process. As the gelatin molecules aggregate into coacervates, they encapsulate the drug. Following cross-linking, the resulting nanoparticles contain the drug, which serves as a vehicle for controlled and targeted drug delivery [70,71].

### 3.3. Emulsion

The emulsion method involves dispersing a gelatin solution in an immiscible liquid, typically an organic solvent, to create an emulsion. This is followed by the evaporation of the solvent, resulting in the formation of GNPs. This method involves creating GNPs with sizes ranging from 100 to 400 nm. It relies on a single water-in-oil (W/O) emulsion. The process involves combining an aqueous phase containing both gelatin and the drug with an oil phase consisting of either an organic solution of polymethylmethacrylate [72] or paraffin oil [69] and then vigorously shaking the mixture. Cross-linking is then achieved through the use of GA [62,63] or genipin [69]. The emulsion method has the advantage of being able to encapsulate both hydrophilic and hydrophobic drugs [73]. However, the use of organic solvents can be a drawback due to potential toxicity and environmental concerns. The choice of solvent, the rate of evaporation, and the concentration of gelatin can all influence the size and properties of the resulting nanoparticles [74].

### 3.4. Nanoprecipitation

**Figure 4:**
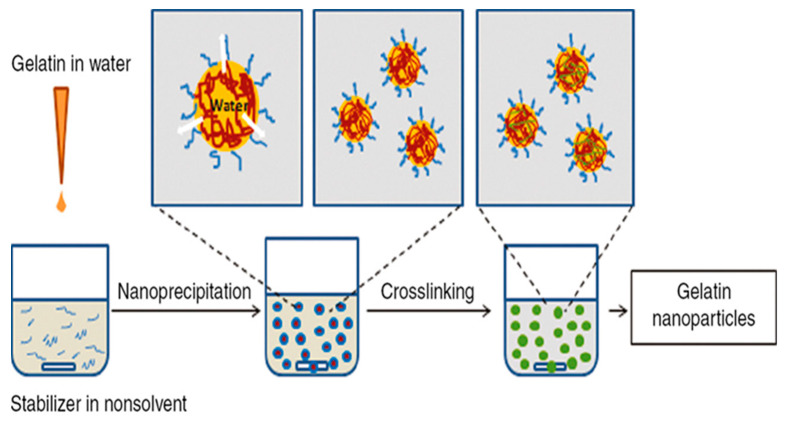
Schematic illustration of the nanoprecipitation method for the preparation of gelatin nanoparticles (reproduced with permission from [77]).

Nanoprecipitation, also known as the solvent displacement method, involves adding an aqueous gelatin solution to an organic solvent, such as ethanol, which contains poloxamer as a stabilizer. Then, GA is added to cross-link the nanoparticles, resulting in the precipitation of GNPs (Figure 4) [75]. This method is relatively straightforward and does not require the use of high temperatures or harsh conditions, making it suitable for encapsulating sensitive and hydrophobic drugs [76]. However, it can be challenging to control the size of the nanoparticles produced by this method, and the use of organic solvents can be a drawback [77].

## 4. Factors Influencing Gelatin Nanoparticle Properties

The properties of GNPs, such as size, shape, surface charge, and drug loading capacity, are critical in determining their effectiveness as drug delivery systems. These characteristics are influenced by various factors during their preparation, including the method of preparation, the concentration of gelatin, the pH and temperature of the solution, and the cross-linking process.

### 4.1. Preparation Method

The preparation technique greatly influences the characteristics of GNPs. As discussed previously, desolvation, coacervation, emulsion, and nanoprecipitation are the most commonly used methods. Each method results in nanoparticles with different characteristics. For instance, the desolvation method may require more steps, including cross-linking to stabilize the nanoparticles and potentially an additional purification step to remove any unreacted cross-linking agent. Coacervation usually produces larger particles compared to nanoprecipitation. Emulsion methods can accommodate both hydrophilic and hydrophobic drugs, influencing the drug loading efficiency and the release profile of the nanoparticles [36].

### 4.2. Concentration of Gelatin

The concentration of gelatin in the initial solution can significantly impact the size and drug-loading capacity of the resulting nanoparticles. A high concentration of gelatin typically results in larger nanoparticles and can also increase the drug encapsulation efficiency. However, it may also lead to the aggregation of nanoparticles, which can be undesirable in certain applications [78].

### 4.3. pH and Temperature

The pH and temperature of the solution during the preparation process can influence the properties of GNPs. Gelatin has an IEP around pH 5, at which the gelatin molecules carry no net electrical charge and tend to aggregate, leading to larger nanoparticles. Similarly, temperature can affect the solubility and conformation of gelatin molecules, which in turn influences the size and stability of the resulting nanoparticles [79].

### 4.4. Cross-Linking Process

The cross-linking process, which stabilizes the GNPs, can significantly impact their properties. The type of cross-linking agent used, the degree of cross-linking, and the duration of the cross-linking process can all influence the size, stability, drug loading capacity, and drug release profile of the nanoparticles [80,81]. For instance, a high degree of cross-linking can increase the stability and control the release of the encapsulated drug, but it may also reduce the drug loading capacity. Understanding how these factors influence the properties of GNPs is essential for tailoring them for specific applications. By carefully controlling these factors, it is possible to produce GNPs with desired characteristics, optimizing their effectiveness as drug delivery systems. Ongoing research in this field continues to provide insights into these relationships, paving the way for the development of more sophisticated and efficient GNP-based drug delivery systems [80,81].

## 5. Optimization of Gelatin Nanoparticles for Drug Delivery Applications

The effectiveness of GNPs as drug delivery systems hinges on their ability to protect the encapsulated drug, transport it to the target site, and release it in a controlled manner. These capabilities are largely determined by the properties of the nanoparticles, such as size, surface charge, drug loading capacity, and release profile [82]. To optimize GNPs for drug delivery applications, these properties can be carefully controlled and tailored to suit the specific requirements of the application.

### 5.1. Size

The size of nanoparticles influences their distribution, cellular uptake, and clearance in the body. Smaller nanoparticles (below 100 nm) are generally preferred for systemic applications because they can evade clearance by the reticuloendothelial system and penetrate deeper into tissues. The size of GNPs can be controlled through the preparation process by adjusting factors such as the concentration of gelatin, the stirring rate, and the temperature [83].

### 5.2. Surface Charge

The stability of nanoparticles, their absorption by cells, and their interaction with biological systems are all influenced by the charge on their surface. GNPs usually have a positive surface charge due to the presence of amino groups in gelatin. This positive charge facilitates their interaction with negatively charged cellular membranes and enhances their uptake. The surface charge can be adjusted by varying the pH during the preparation process or by incorporating charged molecules [78].

### 5.3. Drug Loading Capacity

The drug loading capacity determines the amount of drug that can be delivered per nanoparticle. This can be increased by using a higher concentration of gelatin or modifying the gelatin to increase its interaction with the drug. The drug loading capacity can also be influenced by the drug’s solubility in the gelatin matrix and the compatibility between the drug and the gelatin [84].

### 5.4. Release Profile

The release profile of the drug from the nanoparticles is crucial for achieving a therapeutic effect. Ideally, the drug should be released in a controlled and sustained manner to maintain the drug concentration within the therapeutic window for an extended period of time. This can be achieved by controlling the degree of cross-linking in the nanoparticles, which determines their degradation rate and porosity. Furthermore, by integrating responsive elements into the nanoparticles that react to particular triggers, such as alterations in pH or temperature, the release profile can be adjusted [85]. By understanding and controlling these properties, it is possible to optimize GNPs for various drug delivery applications. Each application may have different requirements; therefore, a one-size-fits-all approach may not be appropriate. Instead, a careful design and optimization process tailored to the specific application is necessary. Ongoing research in this field continues to develop new strategies and techniques for optimizing GNPs, thereby contributing to the advancement of nanoparticle-based drug delivery systems.

## 6. Gelatin Nanoparticles as a Drug Delivery for the Treatment of Infectious Diseases

**Figure 5:**
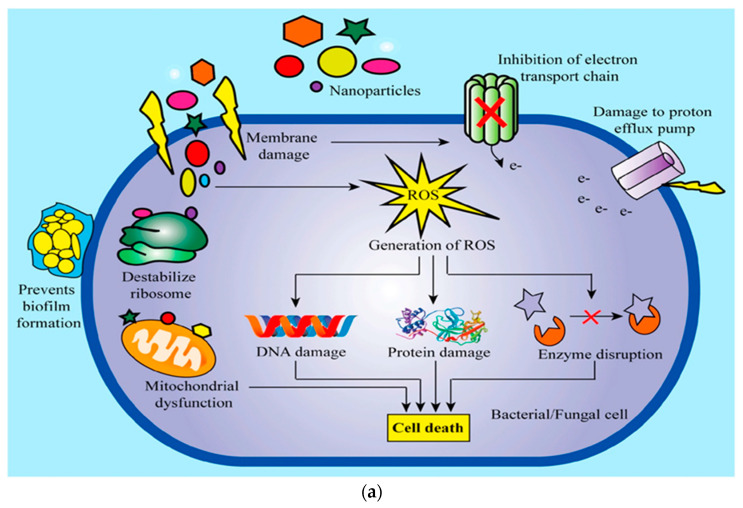

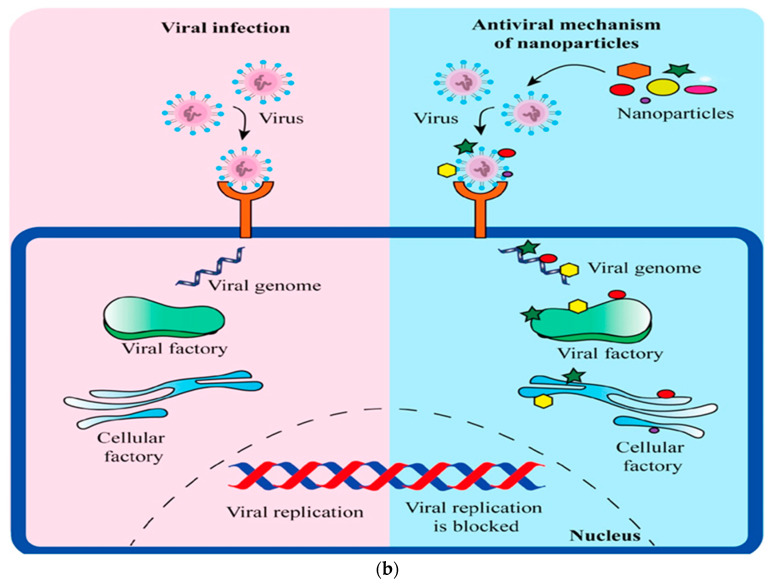
Potential mechanisms of (**a**) antibacterial, antifungal, and (**b**) antiviral drugs delivered by gelatin nanoparticles that can be useful in the treatment of a range of infectious diseases (reproduced with permission from [100]).

GNPs are versatile drug delivery systems capable of encapsulating and delivering a wide range of therapeutic agents, including antibiotics, antivirals, antifungals, and other types of nanoparticles such as metal or carbon nanoparticles (Figure 5). While antibiotics encapsulated in GNPs are still antibiotics and can potentially induce resistance, the encapsulation could enable lower dosages or more targeted delivery, potentially reducing the likelihood of resistance development.

Metal nanoparticles, such as silver nanoparticles (AgNPs), gold nanoparticles (AuNPs), zinc nanoparticles (ZnONPs), and copper nanoparticles (CuONPs), have been extensively studied for their antimicrobial and antibacterial activities [86,87,88,89]. These nanoparticles can cause damage to bacterial cell walls and disrupt essential processes within bacterial cells, effectively killing the bacteria. They have shown efficiency against a wide range of bacterial strains, including antibiotic-resistant strains. However, there are concerns about the potential toxicity of metal nanoparticles to human cells and the environment [90,91].

Carbon-based nanoparticles, such as graphene oxide (GO), have also shown promise as antimicrobial agents [92]. For instance, GO can physically damage bacterial cells by disrupting their cell membranes [93]. It can also produce reactive oxygen species that are toxic to bacteria. Like other nanoparticles, there is ongoing research and discussion regarding their potential toxicity and environmental impact [94].

While the use of nanoparticles can provide novel ways to combat bacterial infections and potentially reduce antibiotic resistance, it is important to note that resistance mechanisms can still develop [95]. For instance, bacteria could develop mechanisms to prevent the attachment of nanoparticles or to efflux them. Therefore, GNPs, with their mentioned properties, can be used as carriers to encapsulate metal nanoparticles, such as silver or gold nanoparticles, which are known for their antimicrobial properties. The resulting hybrid nanoparticles can provide enhanced antibacterial activity and potentially decrease antibiotic resistance by providing an alternative to traditional antibiotic treatments [96].

GNPs have shown promise in the treatment of a variety of infectious diseases. For instance, in tuberculosis, a complex and difficult-to-treat disease, GNPs have been used to deliver first-line anti-tubercular drugs with enhanced bioavailability and reduced side effects [97]. In another study, GNPs were used as a delivery system for the antifungal drug amphotericin B in the treatment of systemic fungal infections. The GNPs enhanced the drug’s stability and reduced its toxicity, enabling higher doses to be administered [98]. Furthermore, GNPs have been utilized for the delivery of antivirals, particularly in the treatment of HIV. The nanoparticles ensure targeted delivery of antiretroviral drugs to the lymphatic system, where HIV primarily resides, thereby increasing the treatment’s effectiveness [99]. The following sections provide a review of how these drugs can be delivered using GNPs.

### 6.1. Antibiotics

**Figure 6:**
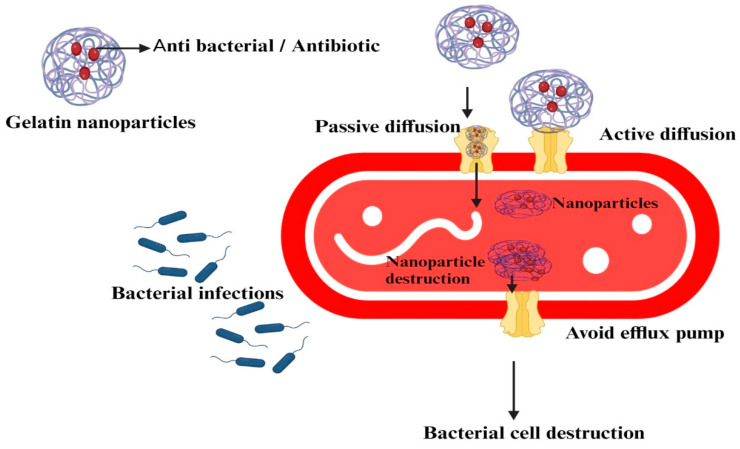
Schematic illustration of delivering antibiotics using gelatin nanoparticles to the site of infection.

GNPs have been extensively used for delivering various antibiotics. They not only protect these drugs from degradation in the physiological environment but also allow for targeted and controlled release, potentially enhancing their effectiveness and reducing side effects (Figure 6) [97]. Several antibiotics, including spectinomycin and chloramphenicol, have been successfully loaded into GNPs for targeted delivery to infection sites, demonstrating enhanced antibacterial activity [36,83].

In the context of bacterial infections, GNPs have been used to deliver antibiotics. For example, one study encapsulated the antibiotic ciprofloxacin in GNPs and found that the encapsulated drug showed enhanced antibacterial activity against *S. aureus* in vitro compared to the free drug. Additionally, the GNPs showed good biocompatibility, suggesting potential for in vivo applications [101]. In order to bypass the immunological elimination executed by macrophages, Li and colleagues [102] presented a strategy involving red blood cell (RBC) membrane-coated, vancomycin-loaded supramolecular GNPs (SGNPs) as a flexible and “on-demand” antibiotic delivery system. As shown in Figure 6, RBC membranes also serve as detoxifying agents that absorb the exotoxins secreted by bacteria, thereby mitigating the inflammation triggered by bacteria. At the same time, the bacterial gelatinases, which are highly expressed in the infectious microenvironment, can efficiently break down SGNPs into minute fragments. This action initiates the release of encapsulated vancomycin, resulting in the local eradication of harmful bacteria. Consequently, this biomimetic antibiotic delivery approach allows for the management of bacterial infections using a minimal dosage of antibiotics [102].

In a study, Ibrahim et al. [83] produced, characterized, and used GNPs for the intracellular administration of weakly cell-penetrating antibiotics, including chloramphenicol and spectinomycin, to improve their antibacterial and antifungal efficacy. These researchers used the desolvation method to synthesize GNPs and then loaded chloramphenicol and spectinomycin into GNPs in addition to cellulosic cotton medical gauze. It was observed that GNPs loaded with these antibiotics, as well as the treated cellulosic cotton gauze, showed an enhanced antibacterial effect against *E. coli* and *S. aureus* [83]. In another study, Schrade et al. [103] developed a dual-stage drug release system for clindamycin and bone morphogenetic protein-2. The system was biodegradable and contained GNPs and hydrogel. During a 28-day period, the release of clindamycin demonstrated a concentration that was 25 times higher than the minimum inhibitory concentration needed to combat *S. aureus* [103].

Fathollahipour et al. [104] prepared and characterized erythromycin-loaded GNPs using the nanoprecipitation method. These GNPs loaded with erythromycins showed antibacterial effects against *S. aureus* and *P. aeruginosa* [104]. Interestingly, Lei et al. [105] synthesized GNPs and subsequently conjugated the antibacterial photothermal peptide AMP-Cypate to create a composite called AMP-Cypate@GNPs. This composite was found to eradicate bacterial infection and promote complete wound healing [105]. In a different study, Mahor et al. [106] developed positively charged GNPs loaded with moxifloxacin for efficient ocular delivery and controlled release in the corneal eye layer. The developed nanosuspension exhibited superior antibacterial activity in vivo against *S. aureus* [106]. *Centella asiatica* extract-loaded GNPs (CGNPs) were developed using desolvation methods. The enhanced antibacterial action against food-borne pathogens was noticed in the case of CGNPs (Table 2) [107].

### 6.2. Antivirals

**Figure 7:**
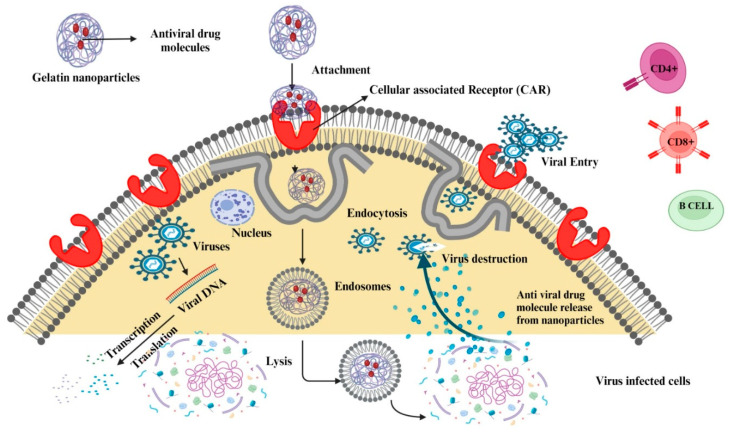
Schematic illustration of delivering antiviral drugs using gelatin nanoparticles to the site of infection.

Antiviral drugs can also be encapsulated in GNPs for improved stability and delivery (Figure 7). For instance, antiretroviral drugs used in the treatment of HIV have been loaded into GNPs, leading to enhanced drug bioavailability and reduced dosing frequency [99]. Similarly, antiviral drugs used for the treatment of hepatitis C have been successfully delivered using GNPs, demonstrating increased therapeutic efficacy and reduced toxicity [25].

Joshy and colleagues [99] developed gelatin-modified lipid nanoparticles as carriers for safe and effective HIV/AIDS therapy. In MCF-7 and neuro-2a brain cells, fluorescence microscopy was used to observe the enhanced cellular internalization of zidovudine-loaded modified nanoparticles. The nanoparticles loaded with the antiviral drug zidovudine showed non-toxicity, sustained release, favorable loading, and hemocompatibility [99]. In a different study, Sabet et al. [25] developed stable GNPs that were conjugated with the non-structural protein 2 (NS2) gene of the hepatitis C virus genotype 4a (HCV4a). This conjugation served as an efficient and risk-free vaccine delivery mechanism. The newly created particles successfully enhanced the transport of genes into bacterial cells without changing the structure of the NS2 gene. As a result, these particles have the potential to be exploited as a non-viral, quick, simple, safe, and inexpensive vaccine delivery technology for HCV [25].

The desolvation procedure was used to create stavudine-loaded gelatin nanoformulations with very low doses, which were then encapsulated in soy lecithin liposomes [108]. Indeed, the formulation exhibited enhanced uptake ability and hemocompatibility with the blood components. Furthermore, this process of stavudine delivery at very low concentrations by means of nanocarriers might offer a novel and effective therapeutic approach for targeting HIV reservoir sites [108]. Rao et al. [109] developed a biomimetic nanodecoy containing a GNP core camouflaged through mosquito medium host cell-derived membranes, which has the ability to entrap the Zika virus (ZIKV) and suppress ZIKV infection. The formulation effectively prevented ZIKV from penetrating physiological barriers into the fetal brain and mitigated ZIKV-induced fetal microcephaly in pregnant mouse models (Table 3) [109].

### 6.3. Antifungals

**Figure 8:**
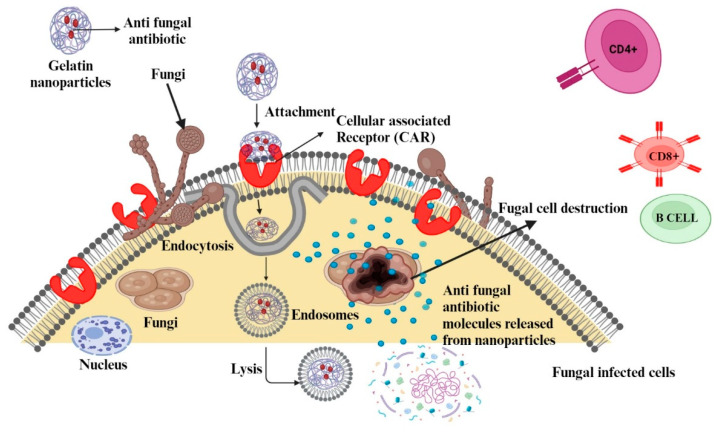
Schematic illustration of delivering antifungal drugs using gelatin nanoparticles to the site of infection.

Antifungal drugs often exhibit poor solubility and bioavailability, and their systemic administration can lead to significant side effects. Encapsulating these drugs in GNPs can enhance their solubility, protect them from degradation, allow for targeted delivery to the infection site, and provide controlled release, potentially improving their effectiveness and safety (Figure 8). For example, antifungal drugs such as amphotericin B and ketoconazole have been successfully delivered using GNPs, showing enhanced antifungal activity and reduced toxicity [110,111].

Ambrosio et al. [112] loaded methylene blue into GNPs and investigated the activity of these loaded nanoparticles on the growth of *Candida albicans*. This study revealed that methylene blue-loaded GNPs enhance the photosensitivity of *C. albicans*, indicating their potential as photodynamic antimicrobial chemotherapy against the growth of *C. albicans* [112]. In another study, Jain et al. [110] developed amphotericin B-loaded polymer-lipid hybrid nanoparticles containing gelatin and lecithin, which were prepared using a two-step desolvation process to ameliorate the oral bioavailability of amphotericin B. In addition to this, the developed formulation showed a sustained drug release profile, enhanced oral bioavailability, and significantly lesser hemolytic toxicity [110]. Ahsan & Rao [111] conjugated anti-TLR4 antibodies onto the surface of ketoconazole-encapsulated GNPs. These GNPs markedly inhibited inflammation, elevated corneal retention, and promoted the resolution of infection in the infected eyes [111]. Aparna et al. [113] developed a sustained-release formulation of amphotericin B-loaded carboxymethyl ɩ-carrageenan conjugated GNPs, which were found to be biocompatible, stable, and non-hemolytic, as well as exhibiting enhanced antifungal activity (Table 4).

## 7. Mechanisms of Drug Delivery Using Gelatin Nanoparticles

GNPs deliver drugs to the site of infection through various mechanisms, which can be broadly categorized into passive targeting, active targeting, and responsive release. These mechanisms enable the targeted and controlled delivery of antibiotics, antivirals, and antifungals, potentially improving drug stability, enhancing drug concentration at the target site, controlling drug release, and reducing side effects [114,115].

### 7.1. Passive Targeting

Passive targeting leverages the natural physiological and pathological traits of the infection site. For example, tissues that are inflamed or infected typically exhibit vascular leakage and inadequate lymphatic drainage, a situation referred to as the enhanced permeability and retention (EPR) effect. Given their small dimensions, GNPs can traverse this leaky vasculature and accumulate at the site of the infection, allowing them to deliver the drug they carry [36].

### 7.2. Active Targeting

Active targeting involves modifying the surface of the GNPs with targeting ligands that can bind to specific receptors that are overexpressed in the infected cells. This allows for the targeted delivery of the drug to the infection site, increasing the drug concentration at the target site and reducing its exposure to healthy tissues. For example, antibiotics, antivirals, or antifungals encapsulated in GNPs can be actively targeted at bacterial, viral, or fungal-infected cells, respectively, by using appropriate targeting ligands [116].

### 7.3. Responsive Release

GNPs can be engineered to release the encapsulated drug in response to specific stimuli at the infection site. These stimuli can include changes in pH, temperature, or the presence of specific enzymes. For instance, bacterial infections often result in a decrease in local pH due to the metabolic activities of the bacteria. GNPs can be designed to release the encapsulated antibiotic in response to the lower pH, providing targeted and timely drug release [117].

### 7.4. Intracellular Delivery

In certain cases, particularly with intracellular infections, it may be necessary for the drug to be delivered into the cells. GNPs, due to their small size and positive surface charge, can be readily internalized by cells through endocytosis [118]. Once inside the cells, the nanoparticles can release the drug, allowing it to act on the intracellular pathogens [118]. Ongoing research in this field continues to explore and optimize these mechanisms, contributing to the development of more effective GNP-based drug delivery systems [53].

## 8. Challenges of Using Gelatin Nanoparticles for Drug Delivery in Infectious Diseases

GNPs have shown immense potential as drug-delivery vehicles in the treatment of infectious diseases. However, their use comes with several challenges that need to be addressed for successful clinical application.

### 8.1. Stability and Storage

GNPs can face stability issues due to their tendency to aggregate, degrade, or prematurely release the encapsulated drug over time or under certain conditions [119]. These stability concerns also apply to storage conditions, where factors such as temperature, humidity, and light exposure can potentially impact nanoparticle stability [120].

### 8.2. Drug Loading and Release Efficiency

The efficiency of drug loading and the subsequent release profile are crucial for the therapeutic effectiveness of GNPs [119]. Achieving high loading efficiency and controlled, sustained release of drugs can be challenging, particularly for hydrophobic drugs or high-dose drugs. Lack of drug penetration is a common problem with nanoparticles, as their slow release and perivascular accumulation can severely interrupt drug delivery [121]. The release profile is also influenced by biological factors, such as pH and enzymes at the infection site, which can be difficult to control [122]. Although the inverse miniemulsion technique used to generate GNPs might provide good loading efficiency for hydrophilic drugs, there are some limitations to consider. Nonetheless, this method results in the generation of polydisperse nanoparticles, which may not be expected where reproducibility is important. In addition, frequent sonication may have a negative impact on some drugs that are loaded, including peptides or proteins. It has been observed that *p*-Xylene removal needs rigorous washing of GNPs, which can also affect their morphology, size, and drug-loading efficiency. As cross-linking agents used in the preparation of GNPs not only result in cross-linking of gelatin but also of therapeutic proteins and peptides, this can further lead to the biological inactivity of these proteinaceous drugs. Therefore, alternative methods are required to avoid the issues associated with cross-linking [123].

Conventional GNP preparation techniques have some limitations in terms of certain drug deliveries, particularly when delivering drugs through the ocular route. The emulsification and micro-emulsification methods require a significant quantity of surfactants in order to generate GNPs, which involves complicated post-processing. The coacervation technique is part of the phase separation process, followed by a cross-linking step. The coacervation technique often results in non-homogeneous cross-linking, which can result in unsatisfactory drug loading efficiency. Moreover, most of the GNP preparation techniques often result in the production of large-sized particles with a high polydispersity index owing to the heterogeneity in the molecular weight of the gelatin polymers. Therefore, a two-step desolvation method was developed to produce GNPs with a decreased tendency for aggregation. This two-step desolvation method is now suggested for GNP preparation since it can overcome numerous drawbacks of conventional preparation techniques and ameliorate upon typical single desolvation technique [124].

GNPs offer biodegradability and biocompatibility; however, they may require chemical modification to achieve prolonged circulation in the physiological environment, which can lead to effective accumulation at the target sites and internalization in cells. Multiple factors can affect the extent of prolonged circulation, including the quantity of protective polymers on the particle surfaces, the molecular weight of the polymers, the lengths of the hydrophobic anchors, and the nature of the polymer used. The shape, molecular weight, and size of the PEG fraction, as well as the type of linkage utilized to attach it to the entity of interest, regulate the outcomes of PEG modification in terms of the adsorption of proteins and various pharmacokinetic properties such as circulation time, volume of distribution, and renal clearance [125].

### 8.3. Scale-Up and Reproducibility

Moving from lab-scale production to mass manufacturing for clinical applications and commercialization presents substantial hurdles. This process needs to be reliable and cost-effective while maintaining the quality attributes of the nanoparticles (e.g., size, drug loading, and stability). Reproducing the same quality of nanoparticles across different batches can be challenging [126].

### 8.4. Biocompatibility and Toxicity

Even though gelatin is generally considered biocompatible and safe, modifications to the gelatin structure (e.g., cross-linking or surface modification) or the encapsulation of certain drugs might introduce safety concerns [127]. The potential immunogenicity of gelatin, along with the toxicity of dissolved cross-linking agents, can pose biocompatibility and toxicity issues [82].

### 8.5. Targeted Delivery and Uptake

Ensuring that the nanoparticles reach the target site and are taken up by the infected cells is another significant challenge [128]. Variations in individual physiology, the presence of biological barriers (e.g., mucus and extracellular matrix), and systemic factors (e.g., immune response and clearance mechanisms) can all impact targeted delivery and uptake [129].

### 8.6. Regulatory Approval

Like any new drug delivery system, GNPs must undergo rigorous preclinical and clinical testing to demonstrate their safety and effectiveness. This process can be time-consuming and expensive and requires careful design and execution of studies to meet regulatory standards [128].

While GNPs offer promising advantages as drug delivery systems for infectious diseases, addressing these challenges will be crucial for their successful translation to clinical use. Ongoing research in nanoparticle engineering, drug formulation, and delivery mechanisms will continue to refine our understanding and use of this promising technology.

### 8.7. Potential Immunogenic Reactions

In general, GA-cross-linked GNPs do not induce unwanted toxicological or immune reactions; however, a lower level of biocompatibility was reported with cross-linked gelatin films. Moreover, oral toxicology studies revealed minor toxicity, as well as the observation of contact dermatitis, when GNPs were applied topically [130]. Since one of the potential future applications of GNPs is their use in humans, substances such as GA have a theoretical residual risk. Therefore, safe alternatives are required. The enzyme transglutaminase is a safe and non-toxic replacement for the chemical cross-linker GA. Microbial transglutaminase is already widely used as a food additive, which covalently cross-links proteins in several processed food industries. Microbial transglutaminase is also used in tissue engineering and has been investigated in GNP preparation. Considering the potential for immunogenicity, the use of a human transglutaminase would be favorable and promising in terms of drug formulation. In this regard, more studies are required with transglutaminase [131].

## 9. Future Perspectives

The field of nanomedicine has been revolutionized by the significant increase in the use of GNPs for drug delivery. These naturally derived, biocompatible, and biodegradable particles have shown promise in delivering a myriad of therapeutic agents, from small-molecule drugs to larger biomolecules such as proteins and nucleic acids. Among the emerging trends in this field are co-delivery systems and stimulus-responsive drug delivery. Co-delivery systems involving GNPs, which are capable of simultaneously delivering two or more drugs, are particularly useful in the treatment of complex diseases that require multi-drug therapy. For instance, co-delivering a chemotherapeutic agent and an immunotherapeutic agent can potentially enhance the efficacy of cancer therapy [132]. Additionally, stimuli-responsive drug delivery systems can release their drug load in response to specific triggers, such as changes in pH, temperature, or the presence of specific enzymes. GNPs that respond to such stimuli are being developed to achieve controlled and targeted drug release, thereby minimizing side effects and improving therapeutic efficacy [133].

Another significant trend in this field is the modification of the nanoparticle surface for active targeting. This approach involves altering the surface of nanoparticles with specific ligands that are capable of binding to receptors on the target cells, significantly enhancing the specificity and efficiency of drug delivery. This trend represents a shift from the traditional focus on passive targeting, which relies on the inherent characteristics of nanoparticles [134]. GNPs are also being explored for their potential use in gene therapy, as their natural positive charge can facilitate the encapsulation and delivery of negatively charged nucleic acids [135]. The combination of gelatin with other nanomaterials to form hybrid nanoparticles is a promising trend. For example, gelatin-coated metallic nanoparticles or carbon nanotubes can combine the advantages of gelatin, such as its biocompatibility and biodegradability, with the unique properties of these nanomaterials, such as their optical properties and electrical conductivity [136]. GNPs are also being studied for their potential use in vaccine delivery. They can protect the antigen from degradation and provide sustained release, thereby enhancing the immune response. This approach is particularly relevant in the development of vaccines for infectious diseases and cancer [137].

There is also a growing interest in photodynamic therapy (PDT) for numerous clinical applications, including the treatment of infections, age-associated macular degeneration, and cancers [138]. PDT involves the use of light-susceptible photosensitizers, which produce singlet oxygen and reactive oxygen species to kill the cells [139]. Phthalocyanines are extremely absorbing organic dyes that are extensively utilized in PDT. Unfortunately, these dyes are hydrophobic in nature and also form aggregates in aqueous systems. Therefore, the use of phthalocyanines might drastically decrease their PDT effectiveness and limit their clinical uses. GNPs hold great promise for ameliorating the efficacy of phthalocyanine photosensitizers utilized in PDT.

Moreover, the versatile properties of gelatin offer options for choosing the most appropriate conditions for the intended drug-release profiles. Studies have already revealed that GNPs can effectively be used to encapsulate photosensitizers or other active drugs [140]. The potential of GNPs in treating infectious diseases is paving the way for numerous intriguing opportunities. With a deepening understanding of biological systems and disease pathology, the development of advanced targeting mechanisms is anticipated. Imagine GNPs adapted to target specific cell types or disease markers, enhancing the precision of drug delivery and reducing off-target effects. This discovery could potentially revolutionize the treatment of infections that remain concealed within specific tissues or cells [116].

The next wave could bring “smart” GNPs that respond to particular physiological conditions or biochemical markers at the infection site. This means more effective treatments with fewer side effects, as drug release could be finely tuned, reducing exposure to healthy tissues [126]. The potential for multi-drug delivery systems is also being explored, particularly for infections that require combination therapies. The simultaneous delivery of multiple drugs could potentially boost treatment effectiveness, combat drug resistance, and minimize the risk of adverse drug interactions [141].

The rise of personalized medicine is another frontier where GNPs could make a significant impact. Tailored nanoparticle systems could be designed based on a patient’s specific needs or genetic profile, allowing for customization of the drug load, release profile, or targeting ligands. This enables highly personalized and effective treatments [142]. The role of GNPs in the next generation of vaccines is also promising. Their ability to protect the antigen, provide sustained release, and potentially act as adjuvants could enhance immune responses and provide better protection against various infectious agents [143].

The concept of theranostics, which combines therapeutic and diagnostic capabilities in one system, is another exciting potential advancement. GNPs could be designed to deliver drugs while also carrying imaging agents, enabling real-time monitoring of drug delivery and treatment response [144]. As the field matures, there may be advancements in the production of GNPs, such as more efficient, scalable, and environmentally friendly production methods. This will be crucial for ensuring that these treatments are widely available and affordable [145].

## 10. Conclusions

In conclusion, the future of GNPs in the treatment of infectious diseases holds immense promise. Potential advances are under consideration that could drastically alter the approach to treating infectious diseases, improve treatment outcomes, and enhance the quality of life for patients. However, to bring these advances into reality, continued investment in research and a commitment to overcoming the technical and regulatory challenges ahead is necessary. The journey is indeed challenging, but the potential global benefits for patients make it an exhilarating pursuit.

## Figures and Tables

**Table 1:** Types of gelatin with pharmaceutical grades and applications. The bloom strength indicates the firmness or softness of the gelatin, where 50 represents the softness and 325 signifies the firmness. The grade of gelatin, including its bloom strength, significantly influences pharmaceutical production as it determines the texture, stability, and dissolution characteristics of the final product.

Grade	Source	Grade (Bloom Strength)	Isoelectric Point (IEP)	Applications	References
Type A Gelatin	Acid-treated collagen, typically from porcine skin or bovine hide	50−125 (low bloom) 175−225 (medium bloom) 225−325 (high bloom)	Around pH 7−9	Used in hard capsules, tablets, coating, and encapsulation of vitamins or supplements	[31,32]
Type B Gelatin	Alkali-treated collagen, typically from bovine bones or fish skin		Around pH 4.8−5	Commonly used in the production of soft gel capsules and suppositories	[32,33]

**Table 2:** List of antibiotics that utilize various methods for the preparation of gelatin nanoparticles for the treatment of bacterial infections.

Type of Infection	Drug Loaded into GNPs	Method of Preparation	Outcomes	References
Bacterial infection	Vancomycin	Desolvation	Enhanced antibacterial activity and allowed the management of bacterial infections utilizing a minimal antibiotic dosage.	[102]
Bacterial infection	Spectinomycin and chloramphenicol	Desolvation	Increased antibacterial effect against *Escherichia coli* and *Staphylococcus aureus*	[83]
Bone infections	Clindamycin	Desolvation	The released concentration of clindamycin was 25 times greater than the minimum inhibitory concentration required to combat *S. aureus*	[103]
Bacterial infection	Erythromycin	Nanoprecipitation	Showed antibacterial effect against *S.* *aureus* and *Pseudomonas aeruginosa*	[104]
Chronic wounds	Antibacterial photothermal peptide AMP-Cypate	Desolvation	Eradiated bacterial infection and led to complete wound healing	[105]
Eye infection	Moxifloxacin	Desolvation	Nanosuspension exhibited superior in vivo antibacterial activity against *S. aureus* as compared to the commercial product	[106]
Food-borne infection	*Centella asiatica* chloroform extract	Desolvation	Showed enhanced antibacterial activity against food-borne pathogens	[107]

**Table 3:** List of antiviral drugs that utilize various methods for preparing gelatin nanoparticles for virus treatment.

Type of Infection	Drug Loaded into GNPs	Method of Preparation	Outcomes	References
HIV infection	Zidovudine	Double-emulsion solvent evaporation	The particles were non-toxic as well as showed sustained release, favorable loading, and hemocompatibility	[99]
Hepatitis C virus infection	Non-structural protein 2 gene of hepatitis C virus genotype 4a	Desolvation	Markedly improved the delivery of the NS2 gene in bacterial cells without disturbing its structure	[25]
HIV-1 infection	Stavudine	Desolvation	The formulation exhibited enhanced uptake ability and hemocompatibility with the blood components	[108]
Zika virus (ZIKV) infection	Gelatin nanoparticle cores camouflaged by mosquito medium host cell membranes	Desolvation	Effectively prevented ZIKV from penetrating physiologic barriers into the fetal brain as well as mitigated ZIKV-caused fetal microcephaly in pregnant mouse models	[109]

**Table 4:** List of antifungal drugs that utilize various methods to prepare gelatin nanoparticles for the treatment of fungal infections.

Type of Infection	Drug Loaded into GNPs	Method of Preparation	Outcomes	References
Fungal infections	Methylene blue	Desolvation	Showed excellent photophysical properties and enhanced photosensitivity of *Candida albicans* to the nanoparticles	[112]
Systemic fungal infections	Amphotericin B	Desolvation	Showed sustained drug release profile, enhanced oral bioavailability, and significantly lesser hemolytic toxicity	[110]
Keratitis	Ketoconazole	Desolvation	Markedly inhibited inflammation, elevated corneal retention, and resolution of infection in the infected eyes	[111]
*Candida glabrata*	Amphotericin B	Desolvation	The formulations were biocompatible, stable, and non-hemolytic, as well as showed enhanced antifungal activity	[113]

## Data Availability

. Not applicable.
